# Peak-ring structure and kinematics from a multi-disciplinary study of the Schrödinger impact basin

**DOI:** 10.1038/ncomms13161

**Published:** 2016-10-20

**Authors:** David A. Kring, Georgiana Y. Kramer, Gareth S. Collins, Ross W. K. Potter, Mitali Chandnani

**Affiliations:** 1 Center for Lunar Science and Exploration, Lunar and Planetary Institute, Universities Space Research Association, 3600 Bay Area Boulevard, Houston, Texas 77058, USA; 2 Impacts and Astromaterials Research Centre, Department of Earth Science and Engineering, Imperial College London, London SW7 2AZ, UK; 3 Department of Geosciences, University of Alaska, Fairbanks, Alaska 99775, USA

## Abstract

The Schrödinger basin on the lunar farside is ∼320 km in diameter and the best-preserved peak-ring basin of its size in the Earth–Moon system. Here we present spectral and photogeologic analyses of data from the Moon Mineralogy Mapper instrument on the Chandrayaan-1 spacecraft and the Lunar Reconnaissance Orbiter Camera (LROC) on the LRO spacecraft, which indicates the peak ring is composed of anorthositic, noritic and troctolitic lithologies that were juxtaposed by several cross-cutting faults during peak-ring formation. Hydrocode simulations indicate the lithologies were uplifted from depths up to 30 km, representing the crust of the lunar farside. Through combining geological and remote-sensing observations with numerical modelling, we show that a Displaced Structural Uplift model is best for peak rings, including that in the *K*–*T* Chicxulub impact crater on Earth. These results may help guide sample selection in lunar sample return missions that are being studied for the multi-agency International Space Exploration Coordination Group.

## Abstract (web summary)

Impact basins on the Moon are considered as the best landing sites for the recovery of information about the lunar interior. To inform future lunar missions, Kring *et al*. combine remote sensing and numerical modelling to generate a geological map of the Schrodinger Impact Basin peak ring.

Uplifted impact basin peak rings can be used to probe planetary interiors. On the Moon, the ∼320 km diameter Schrödinger basin (Fig. 1) is the best preserved basin of its size and has an extraordinary peak ring with which to evaluate the magmatic evolution of the Moon. The mountainous peak ring has a diameter of ∼150 km and rises 1 to 2.5 km above the basin floor, providing an immense cross-section of the deep crust and possibly upper mantle. Exposures of anorthositic, noritic and olivine-bearing (troctolitic) lithologies have been detected with Kaguya, Chandrayaan-1 and Lunar Reconnaissance Orbiter (LRO) data^1^,^2^,^3^. Key to interpreting the lithologies has been to infer the depth from which they were uplifted by the impact. Here we map a portion of Schrödinger's peak ring and then evaluate several kinematic models for its depth of origin.

Schrödinger basin is a complex impact structure centred at 75°S, 132.5°E—near the southwestern rim of the Moon's oldest and largest impact basin, South Pole-Aitken (SPA), where a few kilometres of SPA ejecta^3^, dominated by a mantle component^4^,^5^,^6^, covered farside crust. Schrödinger is believed to be an early Imbrian-aged impact feature (that is, *ca*. 3.8 Ga^3^,^7^,^8^,^9^,^10^). In addition to well-preserved impact-generated materials, the basin floor hosts two younger volcanic deposits^8^,^11^. This makes it a compelling candidate as a landing site for future robotic^12^ and human^13^,^14^,^15^ exploration.

To provide the geologic foundation for interpreting the peak ring, we used Moon Mineralogy Mapper (M^3^), LRO Camera and Lunar Orbiter Laser Altimeter data to generate a detailed geologic map of a representative 1,225 km^2^ portion of Schrödinger's peak ring. The area has relatively gently sloping topography and thus suffers the least from highly contrasting illuminated and shadowed surfaces. These observations were combined with hydrocode simulations of the Schrödinger impact event. This multi-disciplinary study shows that peak-ring material was uplifted from crustal depths before collapsing outward to form the mountainous ring protruding from the basin floor today.

## Results

### Geologic mapping results

The locations of lithologies identified and mapped with M^3^ spectra (Fig. 2a; see ref. 3 for spectroscopic details), and shown in the geological map (Fig. 2b), are supported by half-metre resolution Narrow Angle Camera (NAC) imagery (Supplementary Table 1), which shows they are coincident with boulder fields. The region hosts a series of complex faults that have divided the mapped region into three parts, offset lithologies, and complicated the trace of a large graben that transects the region. The bulk of the regolith that forms the talus slopes of the three major divisions (light grey in Fig. 2b) has rough surfaces at places where the slope grade decreases due to an increased accumulation of regolith. This surface appears less cratered, because most of the craters are erased by slope processes. Isolated patches of smooth regolith can be seen in flat areas (dark grey in Fig. 2b). These places preserve a cluster of fresh craters due to the absence of slope processes.

### Exposed lithologies

Schrödinger's peak ring is the most mineralogically intriguing and complex of the region. The spatial resolution of the M^3^ data used to make the exposed mineral map of Schrödinger's peak ring is 280 m per pixel. The lithologies mapped in Fig. 2b, therefore, represent massive exposures (>78,000 m^2^) of these minerals. In addition, to be identifiable in M^3^ data, and not overwhelmed by the spectrally dominant mineral pyroxene, olivine and plagioclase must modally dominate the area observed in the pixel. The peak ring has three dominant rock types^3^ (Supplementary Fig. 1): a noritic lithology (>10% orthopyroxene+ <90% plagioclase), anorthosite (<10% orthopyroxene+ >90% plagioclase) and a troctolitic lithology (olivine+plagioclase). Anorthosite can be further subdivided into pure anorthosite (>97% plagioclase) and pyroxene-bearing anorthosite (3–10% pyroxene+plagioclase). Although pyroxene can dominate the spectra for anorthosite with <95% plagioclase^16^, spectral features of both are still observable, allowing model proportions to be quantified without a spectral deconvolution model. Such massive accumulations of crystalline material suggest a deep, high-pressure origin, such as the lower crust or upper mantle. The olivine-bearing lithology is troctolite, of inferred crustal origin, rather than dunite, of possible mantle origin. Thus, all of the observed lithologies are consistent with an origin in the crust.

The lithologies tend to occur in isolated hectometre- to kilometre-size outcrops; there are only a few contacts between them. Noritic exposures are isolated in the northwestern quadrant of the study area, constituting roughly two-thirds of the composition of the northern-most division and half of the middle division. Exposures identified as noritic with M^3^ spectra were observed to have the lowest albedo in the NAC mosaic, making it clearly distinguishable from the surrounding lithologies. The noritic peaks *B* and *C* (Fig. 2b) appear to have been separated by the graben 0 and may once have been a part of a larger noritic block. Downslope erosion of the noritic outcrop at summit A, created a trail of scattered boulders and regolith with a noritic signature, which comes to rest against the western edge of troctolitic ridge *D*.

Troctolitic exposures occur on the eastern portion of the northern-most division, run through the centre of the middle division and are seen from east to west all along the north half of the southernmost division. The spectral features of olivine and plagioclase are observed in the troctolitic area in the northern division (*D*). Spectra of the troctolitic area to the south (*E*) are dominated by olivine, such that they lack an observable plagioclase absorption feature. However, the rock is still inferred to contain significant plagioclase, based on the high overall albedo, and because olivine is nonlinearly spectrally dominant over plagioclase^3^. The spectral signature of plagioclase can be seen again in the spectra from the troctolitic ridges labelled *F* and *G* (Fig. 2b), which may have once been a coherent block that was separated by graben 0 (Fig. 2b). Despite the greater number of shadows and weaker spectral signatures in the southernmost division, troctolitic outcrops were observed in small illuminated patches.

Anorthosite occurs mostly as pyroxene-bearing anorthosite and is most abundant on the southern half of the southernmost division, where it was mapped based on albedo in NAC images rather than with M^3^ spectra, because the southernmost division is in shadow in M^3^ data. Pyroxene-bearing anorthosite is also observed as small, discrete outcrops scattered apparently randomly in the study area. Some pyroxene-bearing anorthosite also occurs in a crater east of the peak ring (upper right of Fig. 2b). Pure anorthosite was identified in two relatively small outcrops on the eastern portions of the middle and southernmost divisions.

Where different lithologies are in contact, three different transitions are observed (Fig. 2b): pyroxene-bearing anorthosite to noritic units (*H*) in the northernmost division; pyroxene-bearing anorthosite to pure anorthosite, to olivine-rich troctolitic outcrops (*I*) in the middle division; and troctolitic outcrops to pure anorthosite to troctolitic outcrops again (*J*) in the east of southernmost division. A similar set of juxtaposed lithologies is identifiable in a fresh crater that penetrates a north segment of the peak ring^17^.

In some cases, outcrop lithologies mapped with M^3^ data extend into shadowed areas shown in Fig. 2, because those areas were illuminated when the M^3^ data were collected. Where those outcrops are structurally continuous and have similar albedos in NAC images, the boundaries of the outcrops could also be reliably extended into those shadowed areas.

Some locations indicated as ‘unknown' (brown in Fig. 2b) have been delineated where outcrops could be seen in the NAC mosaic, but the mineralogy could not be identified, because they are in shadow in the M^3^ data and their relative albedo in NAC imagery was ambiguous.

### Structural features

A complicated pattern of faults transected the peak ring when it was emplaced, creating steep cliffs and chasms between vertically offset massifs. These faults juxtapose noritic blocks in the two upper (northern) divisions and troctolitic hills in the middle and lower (southern) divisions. Some of these faults also divide and offset lithologies. Four long faults that are radially aligned with the centre of the basin (1–4; Fig. 2b) divide the peak ring into three parallel ridges in this location. The striking pattern of faults that offset lithologies of the peak ring is reminiscent of the severely faulted central peaks of Earth's Sierra Madera^18^ and Upheaval Dome^19^.

In general, as deep, channel-cutting alluvial erosion is not a post-impact process on the Moon, differential topography such as that seen in the peak ring of Schrödinger must be a primary feature of crater formation and must be produced by faulting. We refer to this as the principle of differential topography of central peaks and peak rings in lunar craters. Some modification may have occurred when impact breccias and melt flowed across the peak ring during emplacement of impact deposits within the crater, but deep and sharply defined differential topography requires faults.

A large, east–west trending graben (0 in Fig. 2b)^3^,^20^ that transects the study area is also radially aligned with the basin centre in this location. To the east, this graben takes an almost 90° turn south so that it trends northwest-southeast and approximately circumferential to the basin centre. The graben can be clearly traced to the east and west, but loses its coherent structure as it intersects the study area due to numerous smaller cross-cutting faults. As it passes through the middle division of the peak ring, the graben offsets exposures of troctolitic and noritic units (Fig. 2b). This type of basin floor fracture has been observed elsewhere on the Moon^21^.

The lithological and structural details of the peak ring are consistent with the collapse of a central uplift wherein material flowed outward, producing nappe-like structures that collided with the inward collapsing walls of the transient crater. The material exposed in that collapsed central structure appear to be crustal in origin (for example, derived from 20 to 30 km depth), although the collapse also appears to have offset material (and thus juxtaposed lithologies) on a kilometre scale. Individual blocks of rock in outcrops of anorthositic, noritic and troctolitic lithologies suggest fracturing and comminution of crustal lithologies on a scale of metres and possibly smaller (the limit of resolution being 0.5 m in the LRO Camera-NAC images). Fragmented rocks with reduced friction and cohesion between those rock fragments would have enhanced flow of the crustal lithologies as the central uplift collapsed.

### Numerical simulation results

To test the interpretation of a crustal origin for the peak ring, numerical simulations of Schrödinger-size impacts were performed using the iSALE hydrocode^22^,^23^,^24^ (which is available at www.isale-code.de). Based on GRAIL gravity data^25^, pre-impact crustal thicknesses of 40 and 20 km were used reflecting the disparate nature of the crust beneath Schrödinger's western and eastern sides, respectively. A nominal impactor size of 25 km was used, which produces an approximately Schrödinger-size basin for a reasonable impact velocity of 15 km s^−1^. Additional model details are provided in the Supplementary Materials.

Regardless of the assumed pre-impact crustal thickness, the simulations show (Fig. 3 and Supplementary Movies 1 and 2) that, following impact, crustal material remains across the floor of the basin, including locations equivalent to Schrödinger's peak ring for both crustal thicknesses. Mantle material is, however, within a few kilometres of the surface in the 20 km case. This is a reflection of the maximum excavation depth, which is ∼19 km for the 20 km-thick pre-impact crust and ∼24 km for the 40 km-thick crust (Fig. 3). Consistent with previous models of structural uplift in lunar basins^26^, material uplifted the greatest distance is not at the post-impact surface (the basin floor), but at a depth equivalent to 0.2–0.35 of the transient crater radius. This difference is a consequence of collapse of the structural uplift: an over-heightened central uplift, without sufficient strength to maintain that uplift against gravity, collapses back into the target, its upper layers spread laterally over the basin floor, becoming thinner. This decreases the relative uplift of these upper target layers compared with deeper parts of the structure that experience little to no outward spreading.

The numerical simulations suggest that crustal thickness modulates the cratering process, because of the density and dynamic strength contrast between the crust and mantle. In the simulations, the strength of the crust and mantle during crater collapse is affected by both thermal softening and acoustic fluidization. Thermal softening is the well-known strength reduction that occurs when rocks are heated, and this weakening can persist for long timescales until the target cools. Acoustic fluidization, on the other hand, is a very transient weakening mechanism, which only persists for a short time until impact-induced acoustic vibrations near the impact site have dissipated and the cold rocks reacquire their static frictional strength. Although the real mechanisms of transient strength reduction in cratering remain unclear, acoustic fluidization is one proposed mechanism that has had considerable success in explaining crater collapse at a range of size scales (for example, see refs 27, 28). In the present simulations, acoustic fluidization is more effective in the cold crust, whereas thermal softening dominates in the warmer mantle, and the overall effect of both mechanisms is a weaker crust than mantle during crater collapse. As a consequence, more deformation is accommodated closer to the surface in the thick-crust simulation compared with the thin-crust simulation (Fig. 3). Thus, the excavation depth is larger (∼24 versus ∼19 km), the uplift of the crust–mantle boundary is larger (∼25 versus ∼15 km) and the maximum structural uplift is smaller (∼40 versus ∼53 km) in the thick-crust scenario.

Owing to the coarseness of the computational mesh, it is difficult to precisely define the width or the centre of the ‘peak ring' in the simulations, but the peak ring in the simulations appears to be slightly narrower and farther from the centre in the thin-crust scenario. This is because a larger volume of stronger mantle rock is deformed in the thin-crust scenario, resulting in a broader, steeper-sided mantle uplift and, consequently, a larger peak-ring diameter than in the thick-crust scenario. We note that the peak ring on the east side of the Schrödinger basin is both qualitatively narrower and slightly farther from the basin centre than on the west side, perhaps reflective of differences in target crustal thickness across the basin. We also note, however, that there is a regional downwards slope towards the east (towards the centre of SPA), and that in the south a pre-existing basin impact structure (the Amundsen–Ganswindt basin) affected peak-ring formation^8^, where it has collapsed completely below the level of the infilling Schrödinger impact melt and breccias. In both the thin- and thick-crust simulations, the topographic summit of the peak ring sits above the edge of the mantle uplift, consistent with recent gravity observations^29^.

To further illustrate the provenance of peak-ring lithologies, the cumulative volume of peak-ring material was computed as a function of depth (Fig. 4). All of the material in the peak ring comes from depths <20 km if the crust was only 20 km thick, whereas that material could come from as deep as 26 km if the crust was 40 km thick. For the purposes of this plot, we defined the peak ring to be that material within 2 km of the surface and between radii of 80–100 km in the thin-crust model and 70–100 km in the thick-crust model, based on the position of the middle of the peak ring in each scenario. Changing those criteria will shift the curves in the plot slightly, but not significantly. Previous work^3^ indicated the Schrödinger target may have been covered with 6 km of SPA ejecta (with slightly more on the east side than the west side), plus another 1–2 km of ejecta from other basins. The provenance of peak-ring material in the hydrocode simulations (Fig. 4) suggests no significant amount of material in the peak ring is SPA ejecta in the 40 km-thick target crust scenario, but up to 15% of the material in the peak ring could be SPA ejecta in the 20 km-thick target crust scenario. No noticeable differences in the distribution of lithologies has, however, been described^3^ between the east and west sides of the basin.

The maximum shock pressure, *P*_s_, seen by material in the peak ring was also recorded (Fig. 5), from which the cumulative volume of peak-ring material that experienced shock pressures in excess of *P*_s_ has been computed (Fig. 6). The peak-ring materials exhibit a range of shock pressures from 10 GPa up to melting (50–80 GPa), but volumetrically they are dominated by <25 GPa materials. The shock pressure of peak-ring materials is lower in the thin-crust case than in the thick-crust case, because the peak-ring materials originate from a greater radial distance, farther from the point of impact. The 25 GPa limit is a useful benchmark, because it is nominally the minimum pressure needed for transformation of (crystalline) anorthite to (glassy) maskelynite^30^ for ≥An_80_ compositions representative of the lunar crust. That minimum pressure increases to 35 GPa for An_20_ compositions, although high pre-impact crustal temperatures may reduce those thresholds^31^. Maskelynite lacks the crystalline structure needed to produce a spectral absorption at 1.25 μm due to electronic transitions of Fe^2+^ in crystalline plagioclase^32^,^33^,^34^. Although we do not discern any areas that can be mapped as maskelynite, up to 5% (20 km crust case) or 30% (40 km crust case) of the massifs could contain maskelynite based on the hydrocode simulations (Fig. 5). The two simulations bracket the actual crustal thickness directly below Schrödinger, so they should bracket the range of shock metamorphism produced in the peak ring (Figs 5 and 6). We also note a small volume of impactor material incorporated in the peak ring, although it is so small that it could not be detected using the spectral analyses used above. The volume and state of this material will depend on impact angle and velocity, and is likely to be exaggerated by the modelling assumption of vertical impact. Three-dimensional simulations of the Chicxulub impact suggest the proportion of impactor material retained in the crater is a strong function of impact angle, decreasing from ∼90% in a vertical impact to <25 and <12%, respectively, for impact angles of 45° and 30° from the horizontal^35^.

The simulations trace the kinematics of material that ends up roughly in the ‘middle' of the peak ring (Fig. 7). Interestingly, although the trajectory in the thick-crust scenario follows a path involving outward excavation, inward and (eventually) upward collapse, followed by outward motion driven by central uplift collapse, the thin-crust scenario is different and arguably consistent with the same source of material identified in a model by Cintala and Grieve^36^, although the motion of that material is different. In this case, the peak-ring material originates from a shallower depth and farther from the impact point. Consequently, it is excavated farther up the transient crater wall, then collapses inward and down initially, before being thrust nearly vertically upward to its final location. The less pronounced outward motion during the last phase of crater modification is a consequence of the fact that the central uplift is almost entirely mantle in this scenario, which is stronger and does not overshoot the target surface to as great an extent as in the 40 km crust scenario.

Because of the rheological difference between the crust and mantle, crustal thickness appears to play a significant role in the amount of overshoot of the central uplift (less for thin crust), the consequent amount of outward motion of the central uplift, the final radius of the peak ring (larger for thin crust), the depth of origin of peak-ring material, and the radial origin, and hence shock pressure of peak-ring materials. In the thin-crust case, the peak-ring materials originate from shallower depths than in the thick-crust case. The cumulative volume of peak-ring material originating above a depth *d*_0_ has been calculated to explicitly show (Fig. 4) that the material in the thin-crust case originates from a shallower depth than the thick-crust case. In both cases, however, the peak-ring materials originate from <30 km depth.

## Discussion

For lunar central peak craters, Cintala and Grieve^37^ pointed out that uplifted impact melt cannot form bedrock peaks, implying the minimum depth of origin for central peaks is the maximum depth of melting. They derived an analytical equation relating structural uplift with final crater diameter^36^. If one applies that equation to Schrödinger, one obtains an estimate for structural uplift of 94 km, which should have exposed material from the lunar mantle.

Schrödinger, however, is a peak-ring basin, not a central peak crater. Cintala and Grieve^36^ suggested an alternative uplift model for structures of this size on the Moon. Rather than having the topographically exposed structure rising from the crater centre, they suggested it rises from a ring of rock bounding the region of impact melted material. In this model, peak-ring lithologies come from shallower depths than the maximum depth of melting. This model is appealing, because it is consistent with the observation of crustal anorthosite, rather than mantle lithologies, in some lunar peak rings^36^. This model has been recently amplified in studies of Orientale^38^ and other basins on the Moon^39^,^40^,^41^, and termed the nested melt-cavity model.

Our simulations of the Schrödinger impact event have, however, two important implications. The peak-ring material is not composed of material that rose from a depth equivalent to the depth of the transient crater (for example, 62.5 km in the case of the model with a 40 km-thick crust; Fig. 8), nor is the peak ring composed of material that was uplifted vertically from the side wall of the transient crater. Rather, it was produced from material in the central uplift that was displaced laterally in nappe-like structrues.

Thus, we prefer to interpret the Schrödinger peak ring with an alternative model, which we refer to here as the Displaced Structural Uplift (DSU) model, wherein central peaks and peak rings are both produced by a similar central uplift process, but in which the central uplift in a larger structure collapses outward and either collides with or overthrusts the inwardly collapsing transient crater rim, to form the peak ring^42^,^43^.

The DSU model for peak-ring basins provides continuity in the processes that produce central peaks (for example, in Copernicus) and peak rings similar to that in the Schrödinger basin. As it generates surface exposures that are derived from depths significantly less than that of the transient crater, it is also consistent with observations of anorthosite in many lunar peak rings, a constraint previously recognized by Cintala and Grieve^36^. For the specific case of the Schrödinger basin, the model implies the lithologies in the peak ring are dominated by crustal lithologies, rather than mantle lithologies.

Although this model treats the process of uplift in peak-ring basins and central peak craters in the same way, one cannot use the previously derived equation^36^ for central uplifts to calculate the depth of origin for material in the peak ring. The collapse of the central uplift in the formation of peak-ring basins alters the amount of final uplift and distribution of lithologies^26^. During the collapse, material in the central uplift flows outward, producing nappe-like structures that collide with the inward collapsing walls of the transient crater. The materials exposed in that collapsed central structure are not the deepest uplifted units, but rather lithologies that are derived from only a fraction of the transient crater depth. That implies a crustal origin for the lithologies within the Schrödinger peak ring, although faulting through the collapsed peak ring could juxtapose and expose units from a range of depths. As noted above, the lithologies observed in the peak ring are consistent with a crustal origin rather than a deeper, mantle origin. If one wanted to interpret the olivine-bearing unit as a mantle dunite rather than a crustal troctolite, it would require more structural offset than implied by the observed faults to juxtapose that mantle lithology with crustal anorthosite, requiring far more mixing than is reasonable.

That finding has important implications for future lunar exploration: samples from the immense and incredibly well-exposed peak ring of Schrödinger basin can be used to derive a cross-section of the lower crust. In addition to the evidence derived from the peak ring, other clues to the structure of the crust occur within Schrödinger basin: normal faults in the terrace zone of the basin expose subsurface lithologies and their stratigraphic relationships, and clasts of subsurface lithologies are entrained in impact melt breccias deposited within the basin and beyond the basin rim. Thus, by combining observations of terrace zones, the peak ring and impact breccias, one can generate a cross-section of the lunar crust that may be tens of kilometres deep. The volume of material beneath an impact site that is melted extends to an even deeper level than the material that is excavated. As that melt is mixed, samples of it will provide an average chemical composition of the crustal volume affected by the impact event. Consequently, a future mission to this site^12^,^13^ could provide a spectacular assessment of the Moon's farside crust.

The DSU model is consistent with hydrocode simulations^44^,^45^ and available observations of the *K*–*T* boundary Chicxulub impact structure^43^,^46^. That model and our observations of the Schrödinger peak ring also have implications for an upcoming International Ocean Discovery Program (IODP) Expedition 364 that is drilling into the buried peak ring of the Chicxulub impact crater. The DSU model suggests peak rings are not simple anticlinal structures that preserve crustal sequences as a function of depth (implied by the nested melt-cavity model), but are instead recumbent fold structures with overturned crustal sequences (Figs 3 and 8). The hydrocode simulations also indicate, however, that the structural and paleodepth sequence seen in a single borehole depends on its radial position on the peak ring. In the outer portion of the peak ring, a single borehole is more likely to penetrate an overturned sequence. In contrast, in the core of the peak ring, a borehole may penetrate an upturned, rather than overturned, sequence. In that case, the core would be composed of units with shallow pre-impact (paleo)depths and then continue into a vertically oriented unit with a deeper, yet relatively constant paleodepth, without completely piercing that unit to re-penetrate the units with a shallower paleodepth. The units in all cases are damaged—fractured with reduced friction and cohesion between those fragments—allowing distortion of the units from different depths, but mixing between paleodepths was not significant at the scale (2 km) of the simulations.

Our observations of the spectacularly exposed peak ring of Schrödinger provide an additional level of lithologic detail not evident in the hydrocode simulations. The kilometre-scale fault displacement exposed at the top of the peak ring of Schrödinger basin (Fig. 2) indicates that material of different paleodepths can be juxtaposed. The observed faults and juxtaposition of lithologies implies one of two outcomes: that the faults are modest modification of the nappe-like structure, and that an overturned sequence at Chicxulub may be evident if the IODP borehole is sufficiently deep. Alternatively, those faults are a near-vertical product of the collision of the outward flowing collapsing peak and the inward flowing modification zone. In this case, one set of faults will have a sense of motion away from the crater centre and another set will have a sense of motion towards the crater centre. Both are listric at depth (as in Fig. 16 of ref. 47). In this case, a borehole will encounter multiple truncating faults rather than an overturned sequence.

**Figure 1:**
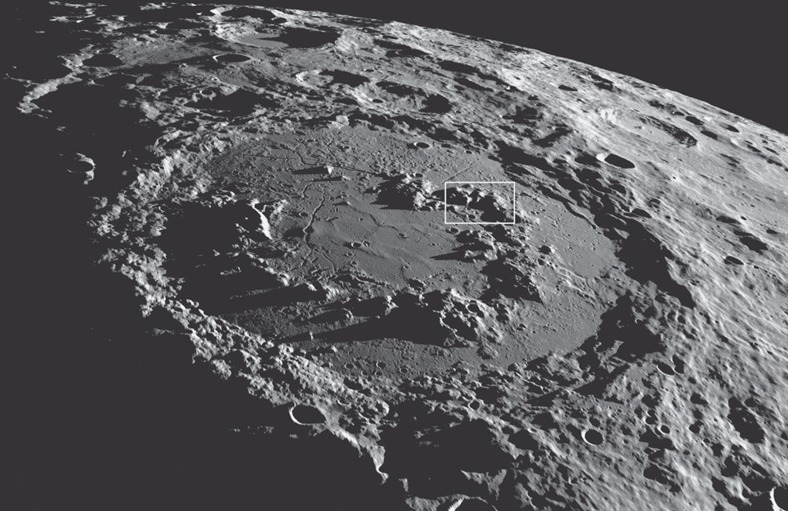
Exposed peak ring. Orbital perspective of the ∼320 km diameter Schrödinger basin on the lunar farside, looking from the north towards the south pole, with a 1–2.5 km-high peak ring rising from the basin floor. The box indicates the area mapped in Fig. 2. NASA's Scientific Visualization Studio. We follow the lunar convention^67^ of referring to this impact structure as a basin, rather than a crater, because it contains a peak ring and has a diameter that exceeds 300 km.

**Figure 2:**
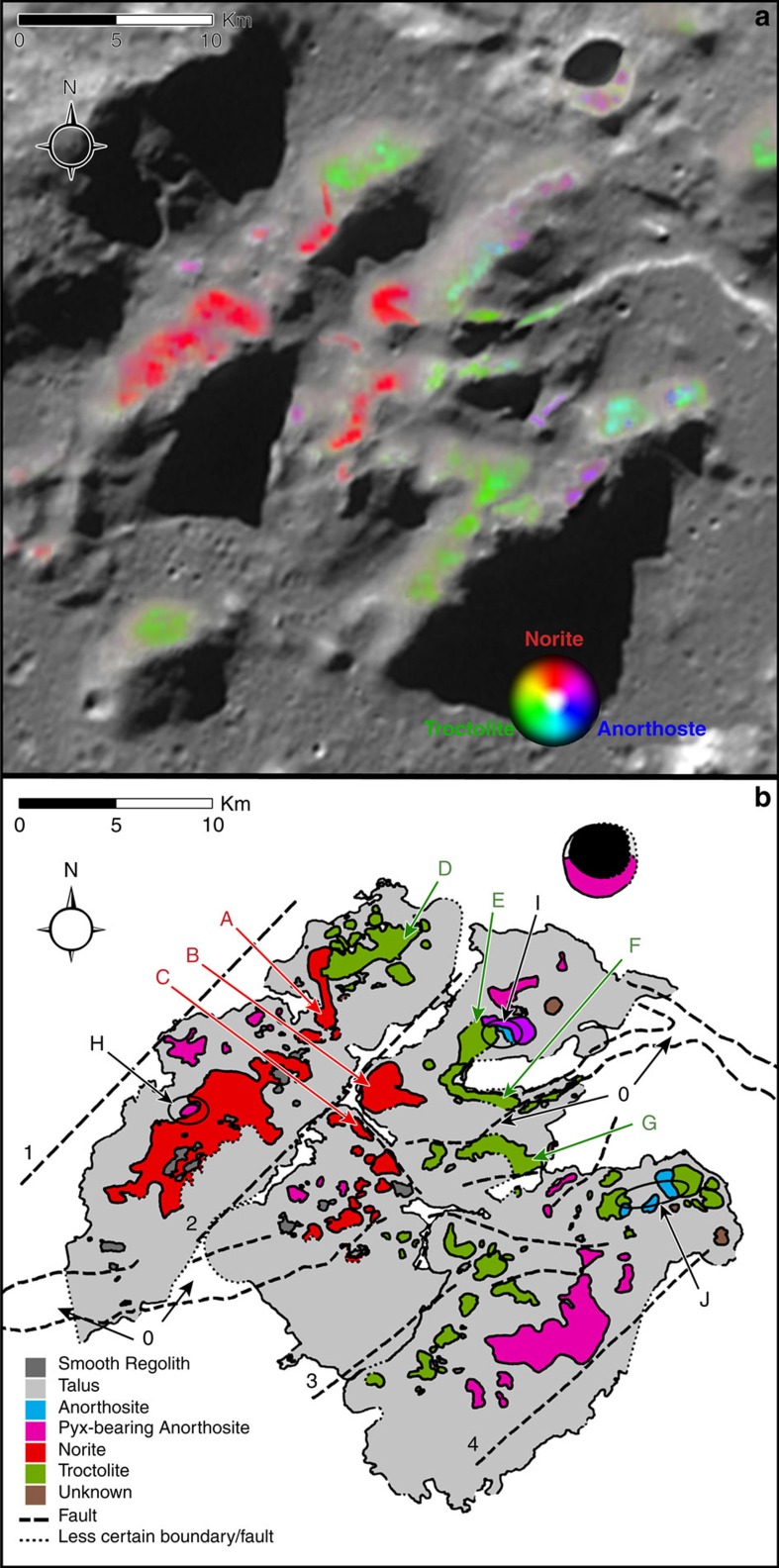
Mapped segment of the impact basin peak ring. (**a**) Lithologies derived from M^3^ spectra draped over an LRO Wide-Angle Camera (WAC) image of the study area. Endmember anorthositic rocks are blue, noritic rocks are red and troctolitic rocks are green; intermediate compositions have intermediate colours. Some areas in shadow in the background WAC image used for context were illuminated when M^3^ spectra were collected. For details of the M^3^ spectral analyses, we refer readers to Kramer *et al*.^3^. (**b**) Geologic map of focus region showing faults, lithological boundaries and talus slope derived by integrating M^3^ results with photogeologic analyses of LRO WAC and NAC images. Letters identify key features: *A*, *B* and *C*=noritic peaks; *D*, *E*, *F* and *G*=troctolitic ridges and summits; *H*=pyroxene-bearing anorthosite adjacent to noritic unit; *I*=transitions from pyroxene-bearing anorthosite to pure anorthosite to olivine-rich troctolitic outcrops; *J*=contacts between troctolitic outcrops and pure anorthositic outcrops. Numbers identify structural elements: 0=graben and 1–4=transecting faults. A key for the color scheme is included on the map. The black area is permanently shadowed, so no images or reflectance spectral data was available. Scale bars, 10 km long (**a**,**b**). The region mapped is bounded by a rectangle with an upper left corner located at 74.7°S, 122.6°E and a lower right corner at 75.7°S, 126.3°E.

It is also important to note that the summits of the massifs in the peak ring of Schrödinger are still fairly sharp (Fig. 1), despite being ∼3.8 billion years old. Regolith formation and mass wasting caused by later volcanic, tectonic and impact events have softened the features, but far less efficiently than erosion on the Earth. Thus, if similar peak-ring summits were produced at Chicxulub, they probably generated colluvial scree on lower slopes and pediments of debris in topographic lows before being buried. It took ∼300 ka before the base of the peak ring was covered with marine sediments^48^; thus, erosion of the peak-ring summits probably occurred over 10^6^–10^7^ years before they were buried. That debris would have been deposited on either exposed target rocks in the peak ring or on top of impact breccias that were previously deposited among the massifs of the peak ring during the impact event such as that seen in Schrödinger^3^,^8^ and implied by breccia deposits that flowed over and beyond the peak ring at Chicxulub^43^,^49^,^50^. Thus, depending on the location of the IODP borehole, lithologies not yet seen in other Chicxulub boreholes may be recovered. The IODP borehole will be an important *in situ* test of the DSU model versus that of the nested melt-cavity model, but interpretations of that borehole will be greatly enhanced by the three-dimensional view of a peak ring provided by the exposures in the Moon's Schrödinger basin.

## Methods

### Geologic mapping

We generated a high-resolution (72 cm per pixel) image mosaic of the target area using data from LRO's NAC. Image numbers are provided in Supplementary Table 1. The NAC data were processed using the USGS Integrated Software for Imagers and Spectrometers^51^,^52^. This involved their conversion to Integrated Software for Imagers and Spectrometers cube files, computation of ground distances and photometry, radiometric corrections, conversion of the strips to map projected files and generation of a seamless mosaic from the cube files. Spectral analysis and creation of the exposed mineralogy map used M^3^ level 2 reflectance data^3^. The mosaics were the basis for identification and mapping of lithological contacts and structural features in the southwestern peak ring using ArcGIS 10 software.

### Hydrocode simulations

Equation of state tables derived using the analytical equation of state package (ANEOS) for granite^53^ and dunite^54^ were used to describe the thermodynamic response of the crust and mantle, respectively. The strength and damage^55^ model parameters were similar to those used in other recent lunar crater studies^26^,^56^. In addition, iSALE uses a constitutive model that accounts for changes in material shear strength that result from changes in pressure, temperature and plastic strain^23^,^57^,^58^. For large crater-forming events, this must be supplemented by some form of weakening mechanism that facilitates deep-seated collapse of the transient cavity^59^,^60^. Although the real mechanisms of transient strength reduction in cratering remain unclear and subject to debate^61^, the weakening mechanism used here is acoustic fluidization^62^. This is implemented in iSALE via the block model^60^,^62^. Choices of parameters for the block model were based on successful models of the Chicxulub impact^28^. Supplementary Table 2 lists all the major parameters used here for modelling of the Schrödinger basin-forming event.

The iSALE simulations of peak-ring basin formation presented here are similar in model design to those of previous studies of the terrestrial Chicxulub impact crater^28^ and larger lunar basins, such as SPA, Imbrium and Orientale^4^,^26^,^56^,^63^,^64^. Here we highlight the major differences in model setup from previous work. For completeness, we provide all important model and material parameters in Supplementary Tables 2 and 3.

A relatively cool thermal profile with depth was used, because Schrödinger is one of the last basins to form. The near-surface thermal gradient was 5 K km^−1^ to a depth of 250 km, with a mantle adiabat (∼1,550 K) below. This is similar to, but somewhat colder than, the cool thermal profile (TP2) used by Potter *et al*.^4^,^56^,^63^; at these temperatures the lithosphere has a high pre-impact yield strength (maximum 700 MPa at 150 km depth). Based on GRAIL gravity data^25^, pre-impact crustal thicknesses of 40 and 20 km were used to reflect the disparate nature of crust beneath Schrödinger's western and eastern sides, respectively. In these simulations, a granite-like material model was used to represent the lunar crust rather than the gabbroic anorthosite material model of Potter *et al*.^4^,^56^,^63^,^64^. This affords the use of an analytical equation of state-derived equation of state table rather than the more simplified Tillotson equation of state for gabbroic anorthosite, and, with reference density of 2,650 kg m^−3^, granite is a closer match to the bulk density of the lunar crust, with almost identical strength properties. Use of this material model also facilitates a more direct comparison with numerical simulations of terrestrial peak-ring crater formation (for example, see refs 28, 65). The acoustic fluidization parameters were chosen based on comparison of simulated crater morphometry to observation for a range of crater sizes (see ref. 66); however, the exploration of acoustic fluidization parameter space was not extensive and these parameters are not regarded as definitive. The effects of dilatancy^28^ were not accounted for in these simulations, but would not dramatically alter the final crater structure or temporal evolution. A nominal impactor size of 25 km was used, which produces an approximately Schrödinger-size basin for a reasonable impact velocity of 15 km s^−1^.

**Figure 3:**
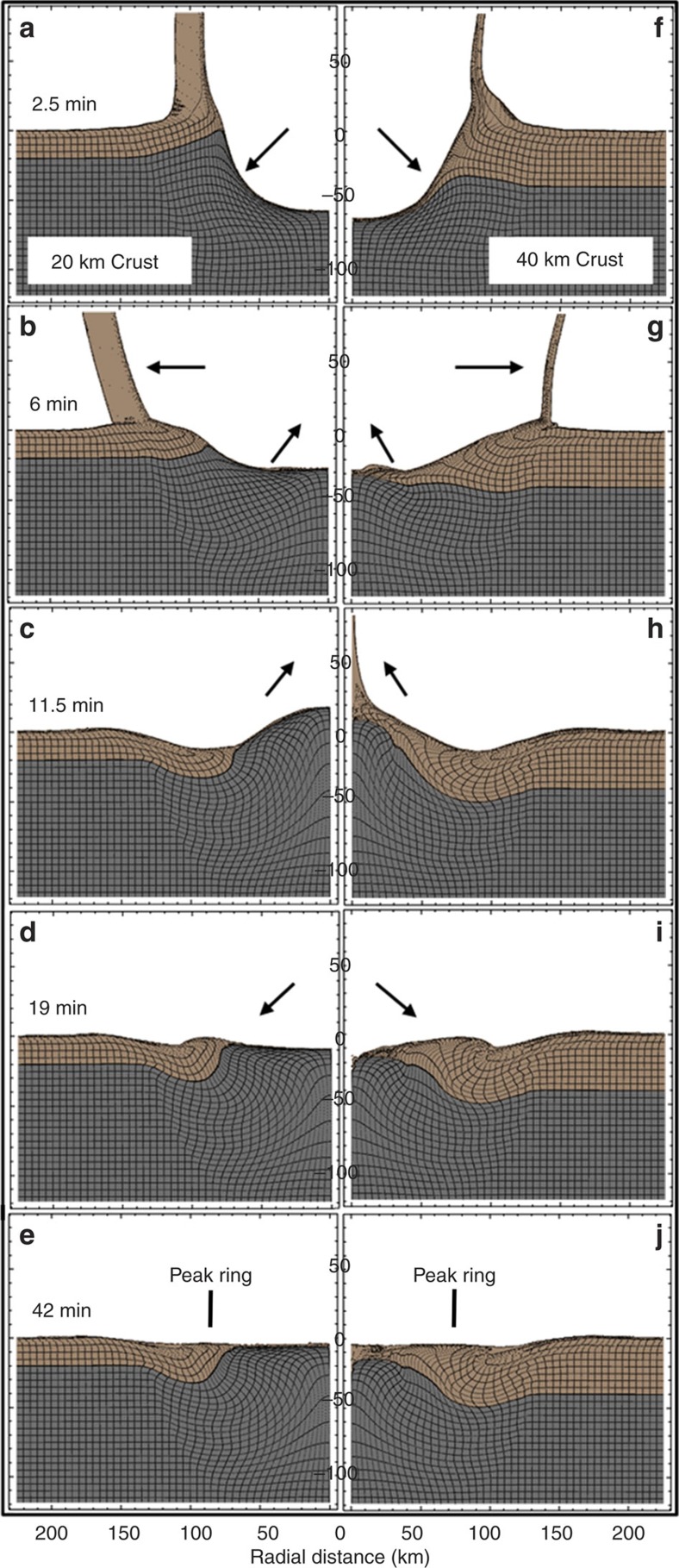
Impact simulations. Five time-steps of the Schrödinger basin impact event modeled using the iSALE hydrocode. The crust is coloured brown and the mantle is coloured grey. (**a**–**e**) Left: the basin-forming event assuming a 20 km-thick target crust, which may better represent the far eastern side of the basin (beyond the study area). (**f**–**j**) Right: the basin-forming event assuming a 40 km-thick target crust, which best represents the side of the basin that produced the massifs in the study area. The cell size is 625 m and there were 20 cells per projectile radius. Arrows highlight the general movement of material during basin formation. (**f**) is shown in a simplified form in Fig. 8a; in **j** is shown in a simplified form in Fig. 8b.

**Figure 4:**
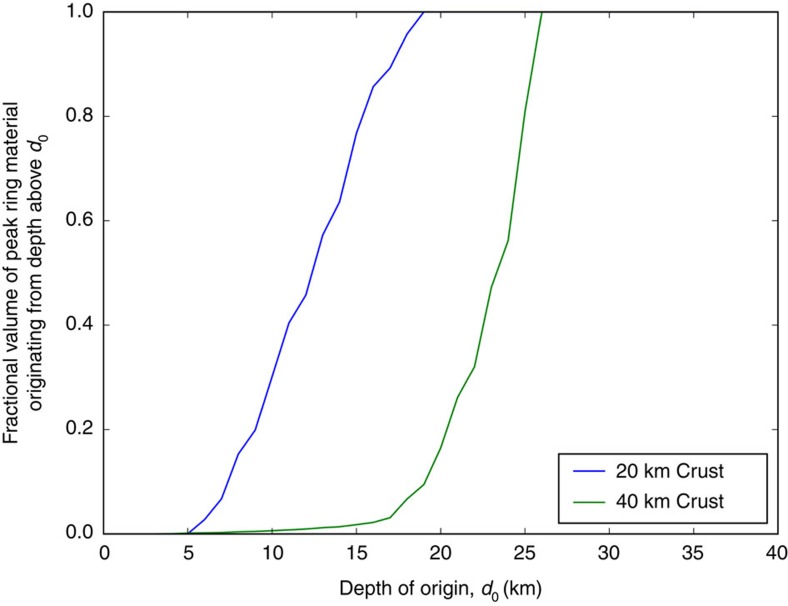
Cumulative volume of peak ring material originating above a depth *d*_0_. In the simulation with a 20 km-thick crust in the target, all of the peak-ring lithologies are derived from depths <20 km. In the simulation with a 40 km-thick crust in the target, some of those lithologies can be derived from slightly more than 25 km depth. Interestingly, we do not see a qualitative difference in the distribution of anorthositic, noritic and troctolitite lithologies across the basin. That is, the same lithologies mapped in Fig. 2 are also seen in other portions of the peak ring^3^, implying that they may occupy a range of depths in the crust.

**Figure 5:**
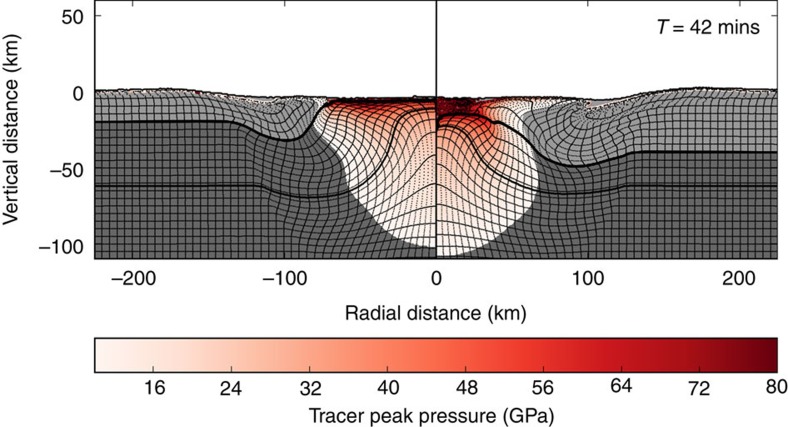
Shock-pressure distribution. Cross-sections with simulation results for a 20 km-thick target crust (left panel) and 40 km-thick target crust (right panel) with maximum shock pressures indicated in a graduated scale from 12 to 80 GPa. Material that experienced maximum shock pressures less than 12 GPa are not highlighted by pressure and are instead highlighted by material, with crust shaded light grey and mantle shaded dark grey.

**Figure 6:**
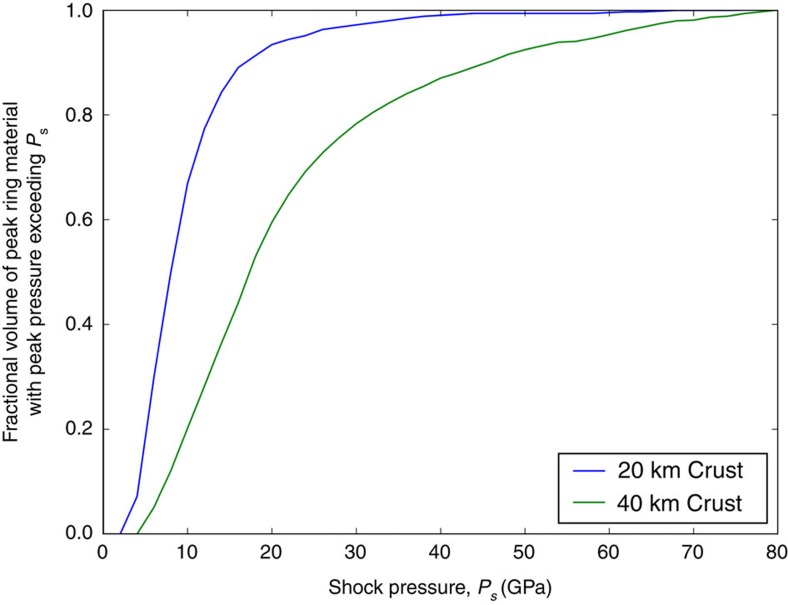
Cumulative volume of peak-ring material that experienced shock pressures in excess of *P*_s_. The results for simulations involving targets with a 20 km-thick crust and 40 km-thick crust are shown. Here the volume of the peak ring is defined as the material within 2 km of the surface, which facilitates comparison with the observed portion of the peak ring and the material that future missions can potentially sample.

**Figure 7:**
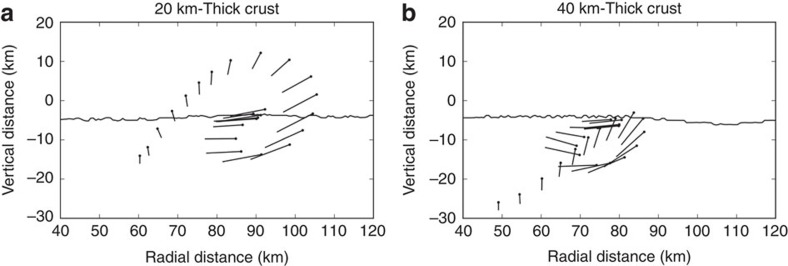
The kinematic flow of material that forms the peak ring. The flow of material is tracked for the (**a**) 20 km-thick crust scenario and (**b**) 40 km-thick crust scenario. The black dot tracks the tracer that ends up closest to the middle of the peak ring; the point at the other end of the line tracks the tracer that starts 2 km below the black dot. Hence, the length and orientation of the line shows the separation and orientation of material that was originally 2 km apart and vertically aligned and becomes stretched and rotated to horizontal through excavation, collapse and uplift.

**Figure 8:**
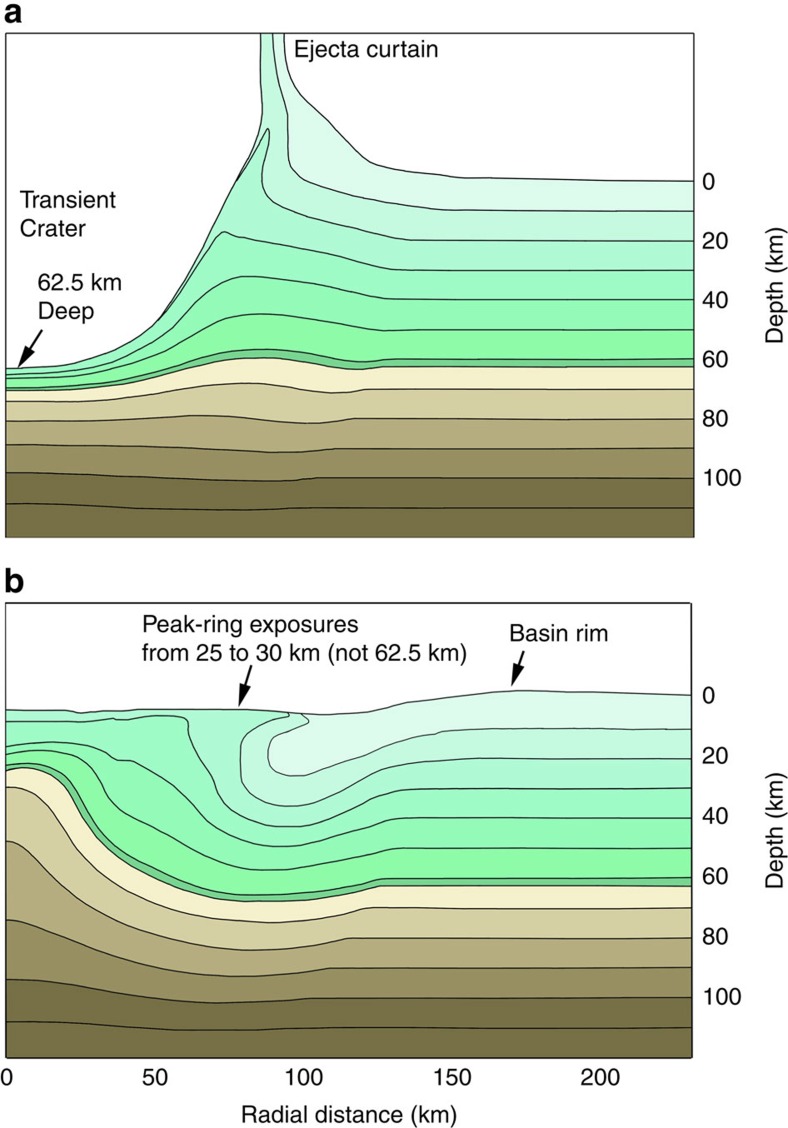
Structural relationships in the Schrödinger basin iSALE hydrocode simulation. (**a**) Time-step at 2.5 min that shows the transient crater depth. It is colour coded to show that relatively shallow units are carried downward to the base of the transient crater. The transient crater radius was 80 km in a target with a crustal thickness of 40 km appropriate for the western side of the basin. (**b**) Time-step at 41.7 min showing the final emplacement and configuration of the peak ring. Results are shaded in 10 km-thick increments and a colour transition between green and brown is used to indicate target units that were stratigraphically above and below the transient crater depth of 62.5 km.

The simulation results, depicted in Fig. 3, compare the large-scale structural evolution of the crust during formation of a Schrödinger basin-scale impact crater for two pre-impact crustal thicknesses (20 and 40 km). Animations based on these two simulations are also provided as Supplementary Materials.

### Data availability

All relevant data are available from the authors on request and/or are included with the manuscript (in the form of data tables or data within figures.)

## Additional information

**How to cite this article:** Kring, D. A. *et al*. Peak-ring structure and kinematics from a multi-disciplinary study of the Schrödinger impact basin. *Nat. Commun.* **7**, 13161 doi: 10.1038/ncomms13161 (2016).

## Supplementary Material

Supplementary Information Supplementary Figure 1, Supplementary Tables 1-3 and Supplementary References

Supplementary Movie 1 Video created by assembling 250 time steps from the numerical simulation of a Schrödinger-size lunar impact assuming a 20 km-thick crust. The time represented by the video is 42 minutes.

Supplementary Movie 2 Video created by assembling 250 time steps from the numerical simulation of a Schrödinger-size lunar impact assuming a 40 km-thick crust, which is suitable for the region of the Schrödinger basin where the peak ring was mapped in this study. The time represented by the video is 42 minutes.
