# Supplementary material for: Peak-ring structure and kinematics from a multi-disciplinary study of the Schrödinger impact basin
Source: Nat Commun. 2016 Oct 20;7:13161. doi: 10.1038/ncomms13161 (PMC5080443; doi:10.1038/ncomms13161)
Supplement: Supplementary Information — Supplementary Figure 1, Supplementary Tables 1-3 and Supplementary References [file ncomms13161-s1.pdf]

**Supplementary Information**

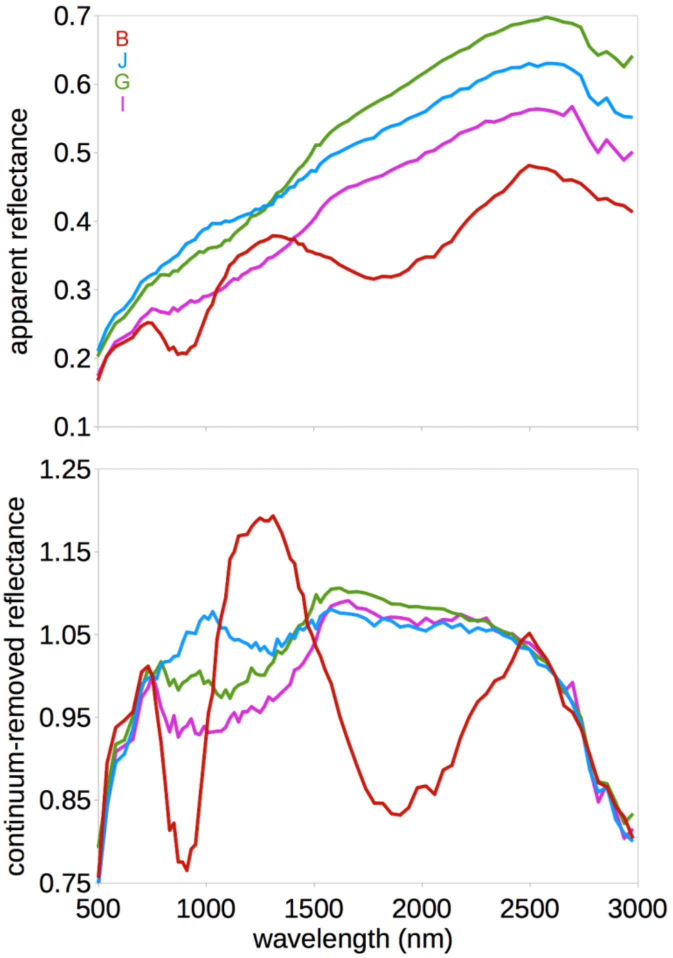


**Supplementary Figure 1.** Representative spectra of the lithologies mapped in Figure 2 Each spectrum is extracted from the M3 mosaic of Schrödinger and keyed to specific locations (B, G, I, and J) in Figure 2. The upper panel provides apparent reflectance and the lower panel show the spectra with the continuum removed using two straight-lines from three points at 750 nm, 1620 nm, and 2540 nm. The orbits used by Kramer et al. (ref. 1) to create the Schrödinger mosaic were obtained between May 28 and July 20 when the Chandrayaan-1 satellite flew at an altitude of 200 km. As a result, spatial resolution of the Schrödinger mosaic is 280 m/pixel.

The NAC images used to map a portion of the peak ring are provided in Supplementary Table 1.

**Supplementary Table 1.** NAC image numbers

M103705161R
M110779249L
M110779249R
M113142422L
M113142422R
M113149226L
M115483764R
M115497357L
M115497357R
M119033407L
M119033407R
M121388109L
M121388109R
M123742885L
M123742885R
M126098507R
M126112085L
M126112085R
M134369197L
M134369197R
M149688598L
M149688598R
M149695385R
M156752360L
M156752360R
M156759141
M156765948L
M156765948R
M159113056L
M159113056R
M161461137R
M167405045L
M167405045R
M167425402L
M167425402R
M174476944
M174483761L
M174483761R
M174497334L
M174497334R
M174504124L
M176840068L
M176840068R
M176860304L
M176860304R

**Supplementary Table 2**. Model parameters used in numerical simulations

| Parameter | Value |
|---|---|
| Impactor diameter (km) | 25 |
| Computational cell size (km) | 0.625 |
| Impact velocity (km $s^{-1}$) | 15 |
| Impactor material | Granite |
| Gravitational acceleration (m $s^{-2}$) | 1.63 |
| Near-surface thermal gradient (K $km^{-1}$) | 5 |
| Lithospheric thickness (conductive thermal gradient; km) | 250 |
| Crustal thickness | 20 / 40 |

**Supplementary Table 3**. Material parameters used in numerical simulations

| **Material parameter** | **Mantle** | **Crust** |
|---|---|---|
| Equation of state type | ANEOS | ANEOS |
| Rock type | Dunite[a] | Granite[b] |
| THERMAL PROPERTIES [c,d,e] | | |
| Solidus temperature (at zero pressure) (K) | 1436 | 1673 |
| Thermal softening parameter | 2 | 1.2 |
| Constant in Simon approximation (Pa) | $1.4\times10^{9}$ | $6\times10^{9}$ |
| Exponent in Simon approximation | 5 | 3 |
| STRENGTH PARAMETERS [c] | | |
| Poisson ratio | 0.25 | 0.25 |
| Cohesion of material (damaged rock) (Pa) | 10000 | 10000 |
| Coefficient of internal friction (damaged) | 0.7 | 0.7 |
| Limiting strength at high pressure (damaged) (Pa) | $3.5\times10^{9}$ | $2.5\times10^{9}$ |
| Cohesion of material (intact rock) (Pa) | $5\times10^{7}$ | $5\times10^{7}$ |
| Coefficient of internal friction (intact) | 1.5 | 2 |
| Limiting strength at high pressure (intact) (Pa) | $3.5\times10^{9}$ | $2.5\times10^{9}$ |
| DAMAGE MODEL PARAMETERS | | |
| Failure strain at zero pressure | 0.0001 | 0.0001 |
| Increase in failure strain with pressure ($Pa^{-1}$) | $1\times10^{-11}$ | $1\times10^{-11}$ |
| Pressure above which failure is compressional (Pa) | $3\times10^{8}$ | $3\times10^{8}$ |
| ACOUSTIC FLUIDIZATION PARAMETERS [f] | | |
| Viscosity scaling factor | 0.015 | 0.015 |
| Decay time scaling factor | 300 | 300 |

[a]ref. 2, [b]ref. 3, [c] Eqs A2 and A3 of ref. 4 for the strength parameters, [d]Table A2 of ref. 5 for the damage parameters, [e]ref. 6 for the Simon approximation,[f]constants in Eqs 11 and 12 of ref. 7.

## Supplementary References


1. Kramer, G.Y., Kring, D.A., Nahm, A.L., & Pieters, C.M. Spectral and photogeologic mapping of Schrödinger Basin and implications for the post-South Pole-Aitken impact deep subsurface stratigraphy. *Icarus* **223**, 131–148 (2013).
2. Benz W., Cameron, A.G.W., & Melosh, J.H. The origin of the Moon and the single impact hypothesis III. *Icarus* **8**, 113-131 (1989).
3. Pierazzo, E., Vickery, A.M., & Melosh, H.J. A reevaluation of impact melt production. *Icarus* **127**, 408-423 (1997).
4. Collins, G.S., Melosh, H.J., & Ivanov, B.A. Modeling damage and deformation in impact simulations. *Meteoritics Planet. Sci.* **39**, 217-231 (2004).
5. Ivanov, B.A., Melosh, H.J., & Pierazo, E. Basin-forming impacts: Reconnaissance modeling. In Reimold, W.U. & Gibson, R. I. (eds.) *Large Meteorite Impacts and Planetary Evolution IV, Geological Society of America Special Paper* **465**, 29-49 (2010).
6. Poirier, J. Introduction to Physics of the Earth's Interior. Cambridge University Press, Cambridge, 264 p. (1991).
7. Wünnemann, K. & Ivanov, B.A. Numerical modeling of the impact crater depth-diameter dependencies in an acoustically fluidized target. *Planet. Space Sci.* **51**, 831-845 (2003).
